# An embarrassingly simple approach for visual navigation of forest environments

**DOI:** 10.3389/frobt.2023.1086798

**Published:** 2023-06-28

**Authors:** Chaoyue Niu, Callum Newlands, Klaus-Peter Zauner, Danesh Tarapore

**Affiliations:** School of Electronics and Computer Science, University of Southampton, Southampton, United Kingdom

**Keywords:** low-viewpoint forest navigation, low-cost sensors, small-sized rovers, sparse swarms, depth prediction, compliant obstacles, forest simulation, off-road navigation

## Abstract

Navigation in forest environments is a challenging and open problem in the area of field robotics. Rovers in forest environments are required to infer the traversability of *a priori* unknown terrains, comprising a number of different types of compliant and rigid obstacles, under varying lighting and weather conditions. The challenges are further compounded for inexpensive small-sized (portable) rovers. While such rovers may be useful for collaboratively monitoring large tracts of forests as a swarm, with low environmental impact, their small-size affords them only a low viewpoint of their proximal terrain. Moreover, their limited view may frequently be partially occluded by compliant obstacles in close proximity such as shrubs and tall grass. Perhaps, consequently, most studies on off-road navigation typically use large-sized rovers equipped with expensive exteroceptive navigation sensors. We design a low-cost navigation system tailored for small-sized forest rovers. For navigation, a light-weight convolution neural network is used to predict depth images from RGB input images from a low-viewpoint monocular camera. Subsequently, a simple coarse-grained navigation algorithm aggregates the predicted depth information to steer our mobile platform towards open traversable areas in the forest while avoiding obstacles. In this study, the steering commands output from our navigation algorithm direct an operator pushing the mobile platform. Our navigation algorithm has been extensively tested in high-fidelity forest simulations and in field trials. Using no more than a 16 × 16 pixel depth prediction image from a 32 × 32 pixel RGB image, our algorithm running on a Raspberry Pi was able to successfully navigate a total of over 750 m of real-world forest terrain comprising shrubs, dense bushes, tall grass, fallen branches, fallen tree trunks, small ditches and mounds, and standing trees, under five different weather conditions and four different times of day. Furthermore, our algorithm exhibits robustness to changes in the mobile platform’s camera pitch angle, motion blur, low lighting at dusk, and high-contrast lighting conditions.

## 1 Introduction

The United Nations Global Forest 2021 sustainability study estimates forests to cover over 31% of landmass, an area of around 4 billion hectares (DESA, 2021). The management, maintenance and conservation of our forests is an enormous operation and of substantial importance to the economy and the environment. A sparse swarm of rovers could assist foresters in monitoring these ecosystems from the ground (Tarapore et al., 2020), gathering spatio-temporal environmental data across vast areas. For example, the swarm could gather census data on healthy tree saplings. It could visually inspect tree barks for symptoms of devastating invasive diseases, allowing identified trees to be precisely managed (Karpyshev et al., 2021). Importantly, to reduce their environmental impact, such as from soil compaction (Batey, 2009), and to allow their large-scale deployment as a swarm, the individual rovers have to be small-sized (portable) and inexpensive.

Off-trail navigation in forest environments remains a difficult task and an open problem in field robotics (Yang et al., 2018). Forest terrain consists of a variety of different vegetation including leaves, twigs, fallen branches, barks, stems, grass, bushes, and creeping vegetation. Rovers are required to predict their traversability over such *a priori* unknown terrains, relying solely on onboard sensors, under varying lighting and weather conditions (Niu et al., 2020; da Silva et al., 2021). Predicting rover-terrain interactions, is consequent not only to the innate characteristics of the forest terrain and the weather conditions (e.g., wet vs dry leaves), but also the dynamics of the interaction between the rover and the terrain which itself is susceptible to change (e.g., long grass tangled around axle of rover, thick layer of mud stuck on rover’s wheels) (Ostafew et al., 2016). The problem is further compounded for small-scale rovers, such as that of a swarm: their small-size affords them only a limited low perspective of their surrounding terrain. Furthermore, consequent to their small size, almost all encountered vegetation is an obstacle, and a small rover is prone to toppling over at obstacles.

Many studies have investigated terrain traversability for navigation in off-road environments (reviewed in Papadakis (2013); Borges et al. (2022)), pioneered by the DARPA PerceptOR (Krotkov et al., 2007) and later the DARPA Learning Applied to Ground Vehicles (Jackel et al., 2006; Huang et al., 2009a; b) and Unmanned Ground Combat Vehicle Perceptor Integration programs (Bagnell et al., 2010; Silver et al., 2010). The majority of these studies discern geometry-based features of the terrain to predict traversability (Santana and Correia, 2011; Santamaria-Navarro et al., 2015; Tang et al., 2019; Haddeler et al., 2020; Lee and Chung, 2021; Wellhausen and Hutter, 2021). For example, Santamaria-Navarro et al. (2015) adopted a time-of-flight camera to determine obstacles online by thresholding the locally estimated normal orientation of queried planar patches of terrain. The authors also trained a Gaussian process model to classify traversable regions offline using terrain slope and roughness features from 3D point cloud data. Similarly, in Lee and Chung (2021), a traversability model was trained using slope, roughness, and curvature features, inferred from eigenvectors and eigenvalues of the covariance matrix of the terrain elevation map. In another example, a large-sized rover in simulation used 3D point cloud data from LiDAR sensors to estimate the gradient of uneven terrain, and consequently quantify the mechanical effort in traversing the terrain Lourenço et al. (2020); Carvalho et al. (2022). Importantly, while geometry-based approaches for terrain traversability have demonstrated some success in navigating rigid terrains such as on well paved paths in structured urban environments (e.g., Bellone et al., 2017; Tang et al., 2019; Liu L. et al., 2020; Lee and Chung, 2021; Lee et al., 2022), they may face potential challenges on compliant terrains such as a forest floor, at a low-viewpoint, with an abundance of grass and other soft vegetation where geometry-based features are unreliable (Haddeler et al., 2022). In such environments, these approaches would potentially result in incomplete elevation maps due to the limitations of the depth sensor hardware.

Geometry-based exteroceptive information are often coupled with proprioceptive information to improve the robustness of terrain analysis (Borges et al., 2022). Here, data-driven near-to-far learning approaches are typically employed to correlate geometry-based features with proprioceptive features such as the rover’s attitude (Murphy et al., 2012; Ho et al., 2013a; Bjelonic et al., 2018; Wolf et al., 2018; Haddeler et al., 2020). The resultant mobility prediction model is commonly used with optimization techniques, such as dynamic programming, to select actions that maximize stability (e.g., see Peynot et al. (2014)). In other studies, geometry-based features are coupled with appearance-based visual features for terrain segmentation and classification (Milella et al., 2015; Schilling et al., 2017; Kragh and Underwood, 2020; Chen et al., 2022; Haddeler et al., 2022). For example, Milella et al. (2015) augmented terrain geometry information from a short-range radar sensor with color and texture information from a long-range monocular camera. The authors used terrain slope information to automatically label traversable and untraversable areas in close-range, that consequently serve as training labels for a long-range visual classifier. In effect, using near-to-far learning, the short-range narrow field-of-view of the radar sensor is extended to the long-range wide field-of-view of a monocular camera.

**FIGURE 1:**
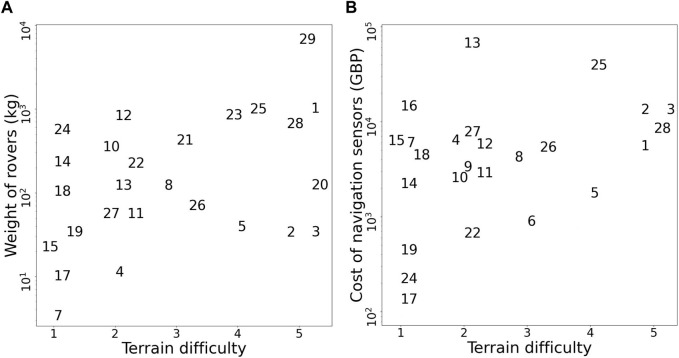
The relationship between terrain difficulty and navigation sensor cost **(A)**, and the weight of the rover **(B)**. Terrains are categorized in ascending order of difficulty as follows: 1. Paved road, short grass; 2. Sandy soil, paved trail; 3. Sparse bushes, tall grass; 4. Rubble, dense bushes, and gravel; and 5. Mars-analogue environment, forest environment–dense bushes, tall grass, fallen branches, fallen tree trunks, standing trees, small mounds and ditches. Importantly, to the best of our knowledge, references 1, 28 and 29 are the only studies investigating navigation in forest environments. All studies referenced are indexed in Table 1.

The existing approaches to off-road navigation appear unsuitable for *inexpensive small-sized forest rovers*. Large-sized rovers, typically used in studies for off-road navigation, perceive the environment from a high viewpoint of around 1 m (e.g., Ho et al. (2013a; b); Ugenti et al. (2022); Fnadi et al. (2020); Baril et al. (2022); Bagnell et al. (2010), also see comparison in Table 1 and Figure 1A). In comparison, for small-sized forest rovers, discerning the forest scene from a size-proportional low viewpoint on the order of centimeters is relatively difficult due to the limited field-of-view in the vertical direction. The field-of-view will frequently be partially occluded by compliant obstacles in proximity, such as grass, leaves, and low-hanging branches. In such scenarios, with large number of proximal compliant obstacles, a fine-grained analysis of the terrain to discern relevant geometric-features may be a waste of computing power. Additionally, geometry-based approaches for traversability analysis commonly use LiDAR or other expensive depth sensors (see Table 1 and Figure 1B), which are not scalable for deployment on large-scale swarms of rovers. Moreover, with appearance-based approaches, environmental factors such as shadows and high-contrast lighting severely affect the visual appearance of the terrain and consequently place high computational demands for feature discrimination (Corke et al., 2013; Ai et al., 2022). Reliable and robust feature discrimination is also sensitive to motion blur, which is more prominent in small-sized rovers due to the unintended tilting and rolling from moving on uneven terrain.

**TABLE 1:** A comparison of terrain traversability studies in off-road environments, with the weight of the rover and the approximate cost of the sensors required for navigation. Sensor costs were obtained from vendor sites, where available. Dashed lines indicate the corresponding data was unavailable.

Index	Reference	Approximate sensors cost (GBP)	Weight of rovers (kg)
1	Ostafew et al. (2016)	3000	900
2	Peynot et al. (2014)	12000	30
3	Ho et al. (2013a), Ho et al. (2013b)	12000	30
4	Bjelonic et al. (2018)	5700	10
5	Krebs et al. (2010)	1600	35
6	Palazzo et al. (2020)	5400	-
7	Murphy et al. (2012)	3800	3
8	Santamaria-Navarro et al. (2015)	800	108
9	Milella et al. (2015)	2300	-
10	Gerdes et al. (2020)	5200	310
11	Mayuku et al. (2021)	2600	50
12	Wolf et al. (2018)	3000	740
13	Kragh and Underwood (2020)	60000	108
14	Ugenti et al. (2022)	2300	206
15	Tang et al. (2019)	4100	20
16	Lee and Chung (2021)	13000	-
17	Sebastian et al. (2019)	122	9
18	Haddeler et al. (2020)	4000	92
19	Wellhausen and Hutter (2021)	400	30
20	Ref in *cf.* Jackel et al. (2006); Huang et al. (2009a,Huang et al. (2009b)	-	109
21	Hoeller et al. (2013)	-	375
22	Liu et al. (2020a)	600	200
23	Braun et al. (2009)	-	750
24	González et al. (2009); Gonzalez et al. (2013)	200	500
25	Fnadi et al. (2020)	35000	880
26	Chen et al. (2022)	4800	62
27	Schilling et al. (2017)	7000	50
28	Baril et al. (2022)	7600	590
29	Howard et al. (2008), Howard et al. (2010); Bagnell et al. (2010); Silver et al. (2010)	-	6000

We propose the design of a low-cost navigation system for small-sized forest rovers, solely using low-resolution depth-prediction images. Our research contribution lies in the uncharted bottom-right region in Figure 1. In our study, a light-weight convolution neural network is used to predict the depth image for the rover from an RGB input image from a monocular camera. A simple coarse-grained navigation algorithm is devised to steer the rover towards open traversable areas in the forest, using mean depth information. Due to additional challenges in designing a high endurance locomotion system for a small-sized low-cost rover, in this study we focus solely on the navigation system. Therefore, our mobile platform is pushed manually by an operator, guided by steering actions on an onboard display. Our developed low-viewpoint navigation algorithm is robust to changes in the camera pitch angle, motion blur, high-contrast lighting, and low-lighting at dusk conditions. It uses a low-resolution 16 × 16 pixel depth prediction image from a 32 × 32 pixel monocular RGB image, and runs on a Raspberry Pi 4. Utilizing low-resolution input images reduces the computational and energy requirements for the rover, enabling efficient navigation for platforms with limited computing capabilities. Our algorithm has been extensively tested in high-fidelity forest simulations and in field trials, successfully navigating a total of over 750 m of real-world forest terrain under five different weather conditions and four different times of day.

## 2 Materials and methods

**FIGURE 2:**
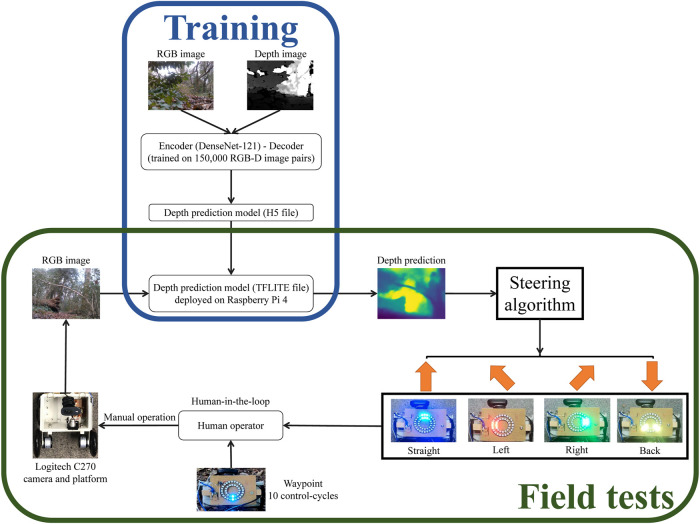
Overview of our approach to forest navigation. During the model training stage, a monocular depth prediction model is trained using RGB-Depth image pairs obtained from the Intel Realsense D435i camera. The model architecture employs an encoder-decoder setup, with the encoder utilizing the DenseNet-121 network and the decoder employing standard convolutional layers. For the subsequent field trials conducted in real-world forest environments, the trained model was converted from an H5 file to a TFLITE file for faster inference. This TFLITE file was then deployed on a Raspberry Pi 4. The deployed model takes input from a Logitech C270 webcam, processes the image, and generates steering actions using a coarse-grained steering algorithm. These steering actions are displayed on LED boards. To execute the generated steering instructions, a human operator interacts with the platform. The operator pushes the platform, following a fixed go-forward displacement and turning angle, and aligns it with the goal waypoint at a fixed interval of every 10 control cycles. The approach combines model training using RGB-Depth images, model deployment on a Raspberry Pi and human-in-the-loop with LED instructions.

Our algorithm for forest navigation comprises of two steps: i) A prediction model is used to infer the depth image from a low-viewpoint low-resolution RGB image input from a monocular camera mounted on our mobile platform; and ii) The output depth image is utilised by our steering algorithm to provide direction motion commands to the operator pushing the mobile platform, to navigate it towards the goal while avoiding obstacles. An overview of our approach for forest navigation is illustrated in Figure 2.

### 2.1 Depth prediction model

Inferring depth from low-resolution RGB images will allow us to use geometric information for navigation, while avoiding the high cost of depth sensors. The depth prediction model has to infer depth in real-time, on an embedded computer, with sufficient accuracy to facilitate navigation of the forest environment. We propose to use a Densenet deep neural network (Huang et al., 2017) for depth prediction, using Densenet as an encoder and following an auto-encoder architecture through transfer learning. In previous studies, Densenet models–Densenet-169 have been successfully used for monocular depth prediction (e.g., see Alhashim and Wonka (2018) trained on both indoor (Nathan Silberman et al., 2012) and outdoor autonomous driving (Geiger et al., 2013) datasets). To the best of our knowledge, no previous studies have validated monocular depth prediction models on unstructured forest datasets.

For our study, the DenseNet encoder pre-trained on the ImageNet dataset (Krizhevsky et al., 2017) was employed. The DenseNet-121 auto-encoder network was then trained on our real-world low-viewpoint forest dataset, comprising a total of 160,000 RGB-D image pairs at 640 × 480 resolution, with ground truth depth in range [0.2 m, 10 m] (Niu et al., 2020). Hyperparameters for training were selected by trial and error using a subset of this dataset, consisting of 5,500 (training), 3,800 (validation), and 1,000 (testing) RGB-D image pairs. During hyperparameter selection, we trained three DenseNet networks–DenseNet-121, DenseNet-169, and DenseNet-201. Consequently, considering the runtime on a Raspberry Pi 4, accuracy, and model size, the Densenet-121, trained for 20 epochs, was selected. The selected model was trained on our entire forest dataset, using 140,000, 10,000, and 8,450 RGB-D image pairs for training, validation and testing, respectively. We ensured that the images in the test set were of an entirely different segment of the forest, compared to the training and validation sets.

The training was implemented using the TensorFlow Keras (Abadi et al., 2016) library on a NVIDIA GTX 1080ti (11G) GPU. An Adam optimizer (Kingma and Ba, 2014) was applied for loss minimization with the default parameters of *B*_1_ = 0.9, *B*_2_ = 0.999, learning rate *λ* = 0.001 and batch size of 8. The loss function was composed of the following: i) Point-wise L1 loss; ii) L1 loss over the image gradient; and iii) Structural similarity loss (for details see Alhashim and Wonka (2018)). During training, data augmentation was applied to reduce over-fitting, including geometric mirroring of RGB images with a probability of 0.5, and photometric swapping of G and R channels from the input RGB images with a probability of 0.5. Note that with our DenseNet-121 model architecture, the resolution of the output predicted depth image was half that of the input RGB image in both dimensions.

We converted the trained Tensorflow model into a TensorFlow-Lite model (Louis et al., 2019), resulting in a three-fold improvement in run-time performance. In estimating the required run-time for our mobile platform, we assume the average speed of a small-sized rover navigating over forest terrain as 0.2 m/s. Additionally, considering the minimum depth range of our ground truth is 0.2 m (Niu et al., 2020), the depth prediction model implemented on an embedded computer with a runtime of about 1 s may be sufficient to achieve real-time performance. Our original 640 × 480 images resulted in a runtime of around 12 s per image on a Raspberry Pi 4. To identify the necessary downscaling, runtimes were tested across several RGB input image resolutions (see Table 2). Since the resolution of 32 × 32 met our real-time requirement, we downsampled the RGB image resolution of our real-world forest dataset from 640 × 480 to 32 × 32, prior to training our Densenet-121 depth prediction model using the same setup. The 32 × 32 Tensorflow-Lite model is 42 MB in size and has a runtime of 0.80 ± 0.02s (mean ± SD) on a Raspberry Pi 4.

**TABLE 2:** Mean ± SD runtime of depth prediction with the DenseNet-121 model, aggregated across 100 randomly selected images, for different input RGB image resolutions. The depth prediction model was executed on a Raspberry Pi 4 (4 GB RAM).

Image resolution	32 × 32	128 × 96	128 × 128	256 × 192	256 × 256	640 × 480
Runtime	0.80 ± 0.02 s	1.35 ± 0.02 s	1.66 ± 0.06 s	2.88 ± 0.02 s	3.60 ± 0.16 s	11.94 ± 0.72 s

**FIGURE 3:**
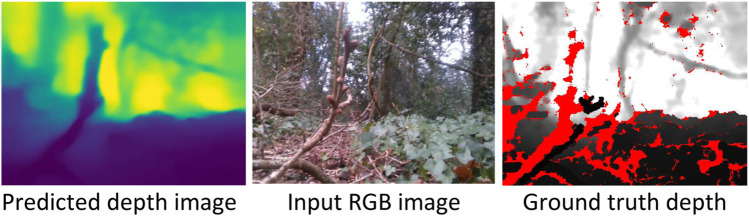
Sample 320 × 240 predicted depth image of a low-viewpoint forest scene from a 640 × 480 input RGB image. Darker (lighter) pixels in the depth image are closer (further away) from the camera. In the predicted depth image, the tree branch in the foreground is distinguishable from the standing trees in the background. The displayed predicted depth image has been upsampled here by a factor of two for visual clarity. Red pixels in the ground truth depth indicate that the depth could not be determined by the Intel RealSense D435i camera.

**FIGURE 4:**
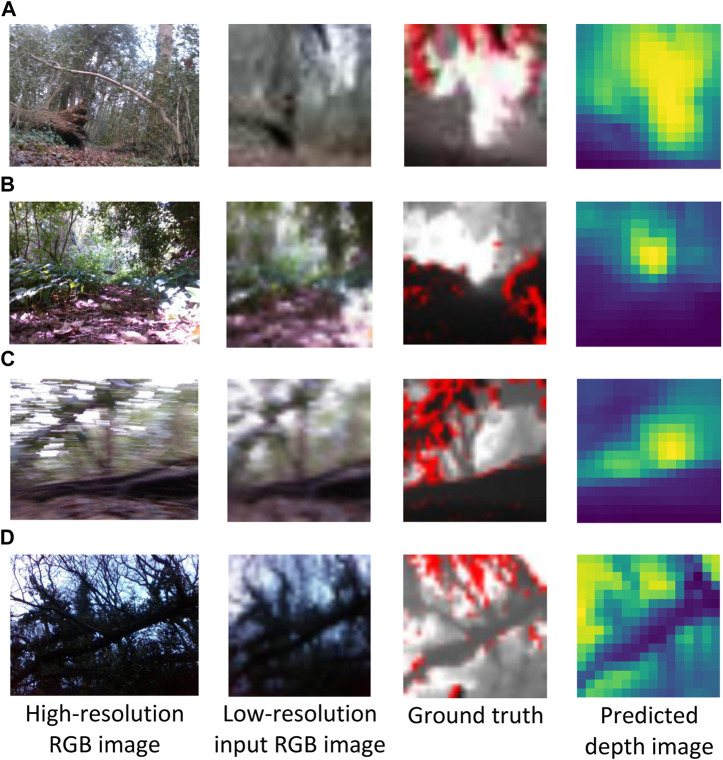
Sample low-resolution 16 × 16 depth images predicted from 32 × 32 input RGB images. The corresponding high resolution (640 × 480) RGB images are shown for context. The depth prediction model was assessed in cloudy **(A)**, high-contrast lighting **(B)**, motion blur **(C)** and low-lighting conditions at dusk **(D)**. RGB and ground truth depth images were captured with an Intel RealSense D435i camera.

We investigate the quality of depth prediction for navigation with high and low resolution input RGB images. With high resolution input RGB images (640 × 480), obstacles in the foreground and background are clearly distinguishable (see example in Figure 3) Despite employing low-resolution RGB images, qualitative results indicate that the depth prediction model can still distinguish between obstacles in the foreground and background (Figure 4). The model appears to provide reasonable predictions in high-contrast scenes (see Figure 4B, the bright and shadowed forest ground in close vicinity have similar predicted depths). As one would expect with the low resolution employed, the results are little affected by motion blur (Figure 4C). Furthermore, obstacles in the foreground and background remain distinguishable in low lighting conditions such as at dusk (see uprooted tree trunk in Figure 4D).

### 2.2 Steering algorithm

Our steering algorithm receives a predicted depth image at every control-cycle, and indicates one of four possible steering directions to the human operator pushing the robot *via* an onboard LED display. The steering directions are “Go-straight”, “Turn-left”, “Turn-right”, “Go-back”, and (orientate towards) “Waypoint”. With the “Go-straight” action the platform is moved straight approximately 50 cm forward. Rotatory actions of “Turn-left” and “Turn-right” pivot the platform by approximately 15° along the yaw axis. Similarly, the “Go-back” action rotates the platform by approximately 180°. Finally, with the “Waypoint” action the platform is rotated towards the direction of the goal waypoint; this occurs here every 10 control-cycles and in general could be based on GPS information.

**FIGURE 5:**
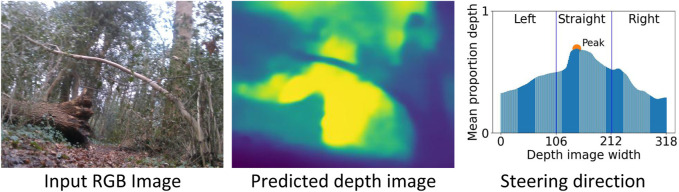
An example steering direction for an input RGB image (640 × 480), the corresponding predicted depth image (320 × 240), and the mean depth averaged across each column of the predicted depth image. As the peak mean depth lies in the central segment of the depth image, the mobile platform is directed straight (towards the traversable region between the fallen tree trunk and the standing tree). The displayed predicted depth image has been upsampled by a factor of two for visual clarity.

For steering, a simple algorithm is used for obstacle avoidance, to select the appropriate action from “Go-straight”, “Turn-left” and “Turn-right” (see example in Figure 5). The predicted depth image is first divided into three equal-sized vertical segments, each corresponding to the “Turn-left”, “Go-straight”, and “Turn-right” actions. Average depth across all the pixels in each column of the depth image is then calculated. The robot is directed to move in the direction associated to the segment with the highest mean depth value. A higher average depth value indicates a region with none or fewer obstacles, corresponding to a relatively more traversable direction. Additionally, averaging over the entire vertical segment allows the steering algorithm to be robust to inadvertent changes in the camera pitch angle during motion.

The “Go-back” direction is used to avoid collisions with close-range obstacles or to avoid encountering large untraversable areas (e.g., a fallen tree trunk) in the distance. It is triggered when the mean lower half of the predicted depth image is less than a predefined threshold of 0.7 m, the LED display then warns the operator of a potential collision. However, false-positives may occur, such as when the robot is on an incline, or if the camera is pointing down towards the ground. Therefore, following the first warning, the operator rotates the robot along the pitch axis by approximately 5°, so that the camera tilts upwards. If the collision warning continues to be displayed, the “Go-back” action is executed. To avoid potential collisions, the above procedure for the “Go-back” action takes priority over all the other steering actions.

### 2.3 Mobile platform

**FIGURE 6:**
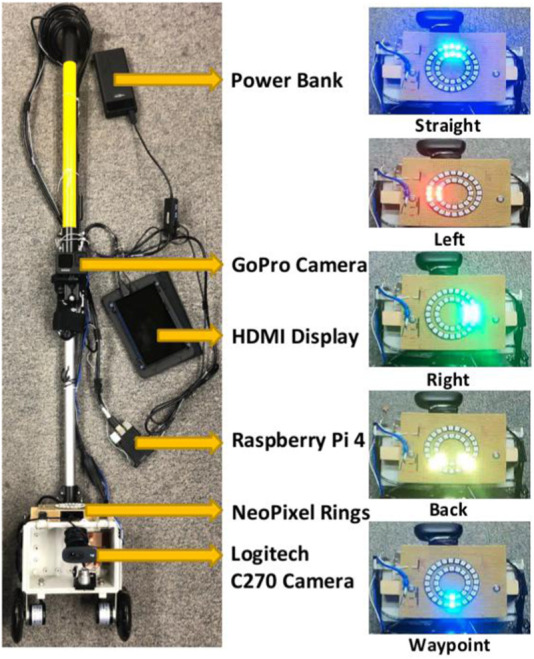
The two-wheeled mobile platform is equipped with a Logitech C270 camera, two NeoPixel rings, a Raspberry Pi 4, a Raspberry Pi HDMI display, a GoPro camera, and a portable power bank. RGB images captured by the Logitech camera are transmitted to the Raspberry Pi 4, to predict depth images for the steering algorithm. Resulting steering actions are displayed on the NeoPixel LED rings.

The mobile platform for our real-world forest experiments (see Figure 6) consists of a telescopic extension pole (1.21 m) and a CamdenBoss X8 series enclosure (L × W × H: 185 × 135 × 100 mm). A Logitech C270 HD webcam (diagonal 55° field of view) is mounted inside the enclosure at 20 cm above the ground and connected to a Raspberry Pi 4. Two black polyurethane scooter wheels, 100 mm in diameter and 24 mm in width, were mounted on the left and right sides underneath the enclosure, to facilitate traversal over rough terrain. A stripboard (95 × 127 mm) was fixed to two rectangular wooden blocks on the top of the enclosure, alongside two concentric NeoPixel rings of addressable RGB LEDs (Adafruit Industries, NY). The two NeoPixel rings were connected to the Raspberry Pi 4 (4 GB RAM) via a twisted pair (data) and a USB cable (power), and a Schmitt-trigger buffer (74LVC1G17 from Diodes Incorporated, TX) in the serial data line was used to overcome the capacitance of the long twisted pair wire. A HERO 9 (GoPro, CA) action camera was also mounted on the telescopic pole 50 cm from the top of the enclosure to allow for third-person view high-resolution video recording of the experiments. The overall cost of our mobile platform was around 250 GBP, with the sensor hardware for navigation—-a Logitech C270 camera and Raspberry Pi 4 embedded computer—costing a total of 70 GBP.

RGB images captured by the Logitech camera every 4 seconds–one control-cycle–were input to the depth prediction model deployed on the Raspberry Pi. Subsequently, the steering algorithm was applied to the predicted depth image, to output a steering direction, which was displayed on the NeoPixel rings (see Figure 6 for details on direction indications). Following the direction displayed, the operator directed the mobile platform to perform the required motion.

## 3 Experiments

Due to the challenging nature of forest field experiments, our navigation algorithm was first tested extensively in simulations (Section 3.1), before investigating its performance on-trail and off-trail in real-world forests (Section 3.2).

### 3.1 Navigating a simulated forest

**FIGURE 7:**
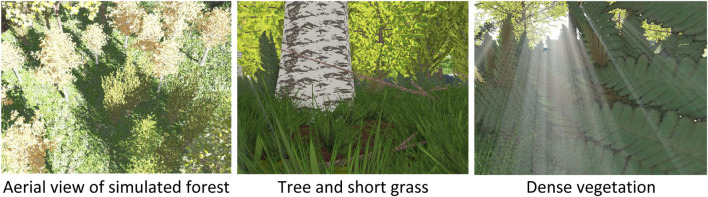
The forest canopy and low-viewpoint RGB images of standing trees and other vegetation from an example forest synthesized by the ForestGenerator (Newlands and Zauner, 2022).

A high-fidelity forest simulator, ForestGenerator (Newlands and Zauner, 2022), was used for the simulation experiments. ForestGenerator is a generalised, open-source tool for generating and rendering interactive, realistic forest scenes using a specialised L-systems, a custom ecosystem simulation algorithm and an OpenGL-based render pipeline and can be controlled from the command line (see Figure 7 for some example rendered scenes). Our simulated rover had the same dimensions as our physical platform. The simulation experiments were performed to investigate the capability of our steering algorithm to navigate the forest environment using low-resolution predicted depth images, in particular discerning how low can we go.

As with our field experiments, for experiments in simulation, a DenseNet-121 depth prediction model was used. The model was trained on a dataset of RGB-D image pairs generated using the ForestGenerator. The scenes comprised different tree species with a cumulative density of around 1 tree/16 m^2^ (see above canopy view in Figure 7). For these images, the simulated camera had a maximum depth range of 10 m. It was positioned at pitch angles of 0° and 30°. For each pitch angle, images were generated from three different viewpoints (0.3 m, 0.5 m and 1 m), each under three different lighting conditions (RGB luminance of 87 ± 22, 99 ± 16 and 118 ± 10 respectively, Mean ± SD across 1000 images). For depth prediction, the DenseNet-121 auto-encoder network pre-trained on the ImageNet dataset was further trained over 20 epochs using 36,000 (training), 2,000 (validation) and 750 (testing) simulated RGB-D image pairs.

Our experiments in simulation were performed in a forest of size 50 × 50 m^2^, with start and goal waypoints at (5 m, 5 m) and (45 m, 45 m) respectively, and with around 150 randomly distributed standing trees. Actions “Go-straight”, “Turn-left”, “Turn-right”, and “Waypoint” (defined in Section 2) were used to navigate the mobile platform towards the goal. For the experiments, the simulated camera was positioned at a low-viewpoint of 30 cm with a pitch angle of 0°, other parameters of the camera were the same as used in the synthetic dataset creation. We assessed the performance of the navigation algorithm using ground truth depth images and predicted depth images in separate experiments for each of six depth image resolutions of 16 × 16, 64 × 48, 64 × 64, 128 × 96, 128 × 128 and 320 × 240, replicated twenty times for each resolution. Therefore, in total 2 sensor-models × 6 image resolutions × 20 replicates = 240 experiments were performed in simulation.

Performance was assessed with the following metrics: i) Total distance traversed by the mobile platform to reach the goal waypoint; ii) The turning rate—the ratio of the number of “Turn-left” and “Turn-right” steering actions over the total number of actions—is zero for a straight-line trajectory and in general unbounded (arbitrary long detours and arbitrary many turns without forward progress); and iii) The number of collisions sustained. In case of a collision, the platform was repositioned next to the obstacle.

**FIGURE 8:**
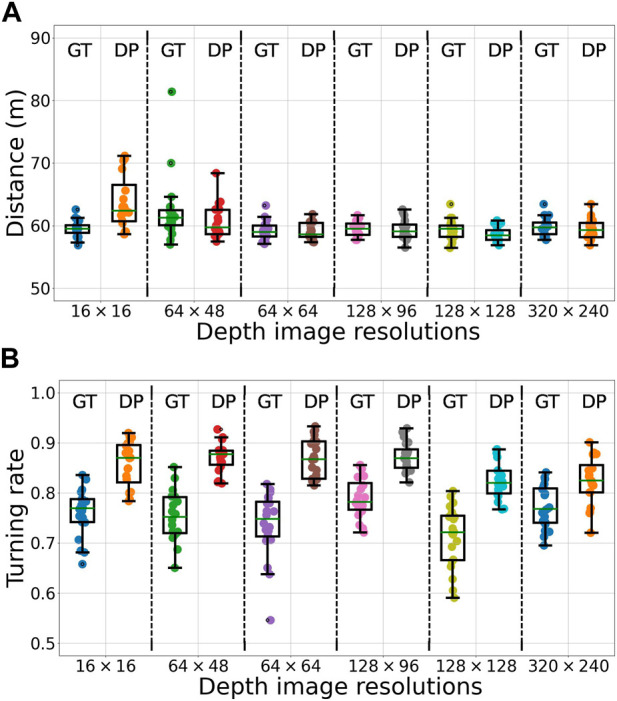
The distance traversed **(A)** and the turning rate **(B)** from start-to-goal for the mobile platform in simulated forests, across 20 replicates, in each of the 12 combinations of sensor-models—ground truth (GT) and predicted depth (DP)—and depth image resolutions—16 × 16, 64 × 48, 64 × 64, 128 × 96, 128 × 128 and 320 × 240. On each box, the mid-line marks the median, and the box extends from the lower to upper quartile below and above the median. Whisker outside the box indicate the maximum and minimum values, except in case of outliers, which are shown as dots. Outliers are data points outside of 1.5 times the interquartile range. Note that the *y*-axes do not start at zero.

In all simulation experiments, the mobile platform was able to arrive at the goal waypoint. With the ground truth depth image, the steering algorithm was able to efficiently navigate the platform irrespective of the input image resolution, traversing a mean distance of 59.95 ± 1.2 m to reach the goal (across all six input image resolutions, see Figure 8A); the straight-line distance between start and goal is 56.57 m. The performance of the steering algorithm was almost constant across several different resolutions of the predicted depth image 64 × 48, 64 × 64, 128 × 96, 128 × 128 and 320 × 240 (mean traversed distance 59.45 ± 0.7 m). However, with the lowest resolution predicted depth images of 16 × 16 steering was less efficient, with the mobile platform traversing a distance of 63.68 ± 4.4 m to reach the goal.

Similar trends in performance were observed for the turning rate of the mobile platform (see Figure 8B). With the ground truth depth images, the turning rate was not influenced by the resolution, at 0.75 ± 0.02 across all six input image resolutions. The performance deteriorated to 0.85 ± 0.02 with predicted depth images.

The slightly poor performance in the distance traversed and the turning rate for low-resolution predicted depth images is ascribed to the coarse-grained prediction of depth of background obstacles. When obstacles in the foreground appeared few to none, this results in a higher uncertainty of steering action. Unable to discern narrow traversable gaps between trees in the background, the mobile platform may be directed to a detour around instead of in between the trees. In such scenarios, a potential solution may be to weigh the inferred steering direction towards the direction of the waypoint. Importantly, in navigating the simulated forest environment, the mobile platform sustained no collisions in almost all the replicates. In particular, at a 16 × 16 resolution, a single collision was sustained in each of three replicates, with no collisions sustained in the remaining 17 replicates (see Supplementary Table S1 in Supplementary Material).

In summary, rather than requiring a precise depth value for each pixel, our steering algorithm relies only on whether the foreground and nearby obstacles are visually distinguishable from the background. As such, our navigation algorithm is largely tolerant to inaccuracies in depth prediction. The somewhat lower performance of the navigation algorithm with a 16 × 16 resolution predicted depth image is largely offset by the high run-time performance, thus supporting its use for our real-world forest experiments.

### 3.2 Field experiments

Our field experiments were performed in the Southampton Common (Hampshire, UK), a woodland area of approximately 1.48 km^2^. Experiments were performed in the following two sites of the Common: i) Following a long forest trail; and ii) Steering through a smaller but more challenging off-trail forest environment.

**FIGURE 9:**
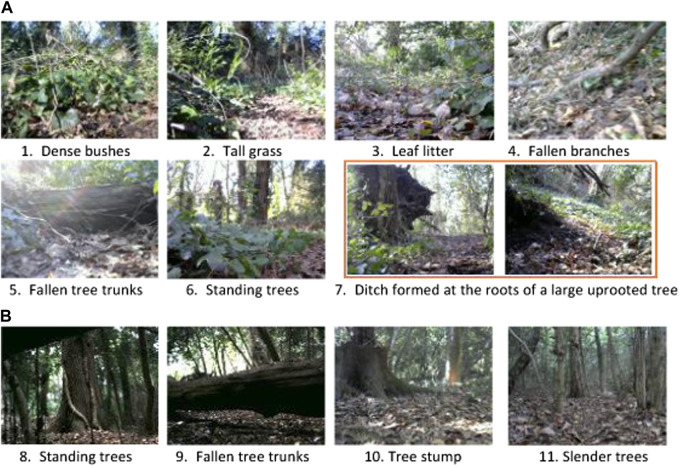
Examples of obstacles encountered by the mobile platform both on the forest trail **(A)** and off-trail **(B)** in the Southampton Common woodland.

For the field experiments, besides the trajectory length, turning rate and number of collisions sustained, the following additional metrics were used to assess the performance of the navigation algorithm in navigating from start to goal waypoints: i) Time taken to reach the goal; ii) Number of true-positive incidents—the “Go-back” action is accurately triggered on encountering a large obstacle (e.g., fallen tree trunk) blocking the path of the mobile platform; iii) Number of false-positive incidents—the “Go-back” action is unnecessarily triggered, i.e., there are no obstacles obstructing the platform; and iv) Number of false-negative incidents—the “Go-back” action is not triggered in the presence of a large obstacle obstructing the platform, thus risking a potential collision. In total, seven metrics were used to assess navigation performance in our field experiments.Following a long forest trail. The mobile platform was tested on a dried mud trail comprising various forest obstacles. Obstacles included dense bushes, tall grass, leaf litter, fallen branches, fallen tree trunks, standing trees and a ditch formed at the roots of a large uprooted tree (see examples in Figure 9A).

**FIGURE 10:**
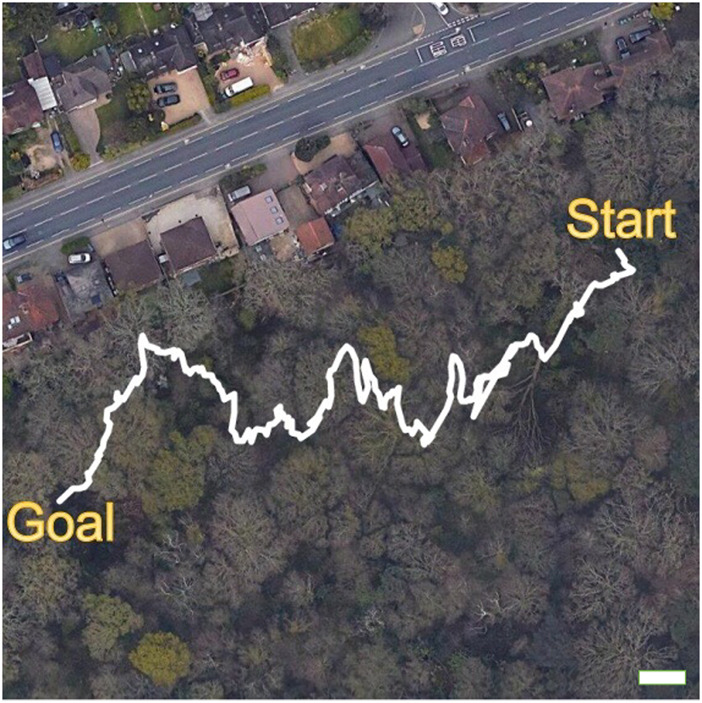
Trajectory from GPS metadata of the forest trail overlaid on an aerial view of the Southampton Common woodland. The white scale bar in the lower right corner corresponds to a distance of 10 m. Permitted use: Imagery © 2022 Getmapping plc, Infoterra Ltd & Bluesky, Maxar Technologies, The GeoInformation Group, Map data © 2022 Google.

For our experiments, the start and goal waypoints were positioned at (5056.2141 N, 124.0516 W) and (5056.1859 N, 124.1515 W), respectively (see Figure 10). The waypoints were selected to encompass a high diversity of compliant and rigid forest obstacles such as leaf litter, twigs, fallen branches, fallen tree trunks, standing trees, grass, bushes, and creeping vegetation (see examples in Figure 9A). The actions “Go-straight”, “Turn-left”, “Turn-right”, “Go-back” and “Waypoint” (defined in Section 2) were used to navigate the mobile platform towards the goal waypoint. As the goal was 210° SW of the start location, this bearing was used to rotate the mobile platform to face the goal when the “Waypoint” action was triggered. The “Go-back” action was employed by the mobile platform to turn around and attempt to find an alternative path to circumvent large obstacles such as fallen tree trunks. If this action was triggered five times consecutively for the same obstacle, we assumed that there were no traversable paths around the obstacle; consequently, the operator would lift the platform over the obstacle, log the incident, and continue the experiment.

**FIGURE 11:**
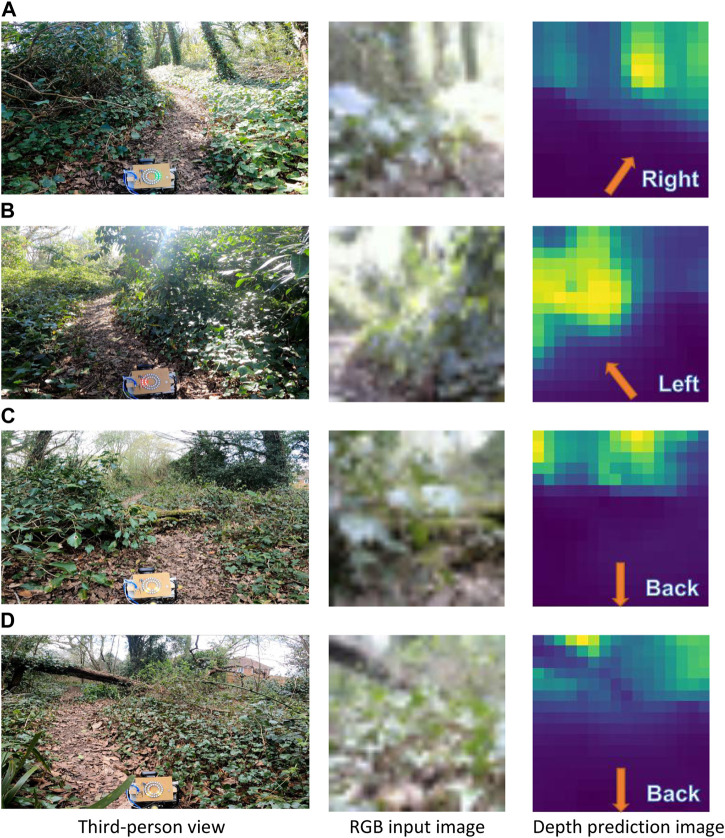
Steering directions navigated by the mobile platform on the forest trail at the Southampton Common woodland, including for clear trails and the right and left **(A, B)**, a fallen tree trunk covered in weeds and moss **(C)**, and dense vegetation **(D)**. For steering, 16 × 16 depth images, predicted from 32 × 32 RGB input images, were utilized. The corresponding 1920 × 1080 RGB images (from the GoPro camera), display a third-person view of the forest scene and the steering commands on the LED rings of the mobile platform. The steering directions are annotated on the predicted depth image. The predicted depth images displayed here have been upsampled here by a factor of two for visual clarity.

Trail experiments were performed five times in the forenoon, midday, and afternoon under weather conditions of cloudy, scattered clouds, mostly clear, and sunny (see details in Supplementary Table S2 of the Supplementary Material). Across all experiments, the platform was able to reach the goal waypoint, traversing a mean distance of 146 ± 3 m with a turning rate of 0.53 ± 0.05 in 20.7 ± 4.9 min (see Table 3). Our algorithm was largely able to steer the platform towards open spaces to avoid potential collisions (see examples in Figures 11A,B; for additional examples of steering by low-hanging tree branches, tall grass, and fallen tree trunks see Supplementary Figure S1 and the demonstration video of the Supplementary Material). In scenarios where the robot was facing a close-range obstacle, or large untraversable areas in the distance, the “Go-back” action was successfully triggered to avoid potential collisions (see Figure 11C—a fallen tree trunk covered in weeds and moss,; Figure 11D—dense bushes). The “Go-back” action was unnecessarily triggered only once, i.e., a false-positive incident, when the platform was facing uphill in a small ditch. Importantly, across all five experiments, the robot navigated the forest trail without sustaining any collisions, or incurring any false-negative incidents. Finally, once in each of the five experiments, due to a large fallen tree blocking the forest trail, the platform had to be lifted over it, as no traversable paths were found to circumvent the obstacle.

Off-trail forest navigation. Experiments were performed in an unfrequented area of the woodland, spanning around 400 m^2^. Obstacles on the site included forest litter, small shrubs, mounds, standing trees, fallen branches and fallen tree trunks (see examples in Figure 9B); the site was more cluttered than the forest trail environment. For our experiments, the start waypoints were located at (5056.1906 N, 124.0071 W) in site A and (5056.1814 N, 124.0148 W) in site B, in two separate and independent setups, with a common goal waypoint at (5056.1853 N, 124.0143 W).

**TABLE 3:** The trajectory length, turning rate, traversal time and the number of true-positive incidents—the “Go-back” action is accurately triggered on encountering an obstacle—in following a forest trail from start-to-goal in the Southampton Common woodland. The experiment weather conditions and times of day are detailed in Supplementary Table S2 of the Supplementary Material.

	Trajectory length m)	Turning rate	Traversal time (mins)	True-positive “Go-back” actions
Run 1	146	0.55	23.9	7
Run 2	149	0.58	27.2	11
Run 3	150	0.54	18.7	9
Run 4	144	0.45	14.7	9
Run 5	143	0.54	18.9	10

**FIGURE 12:**
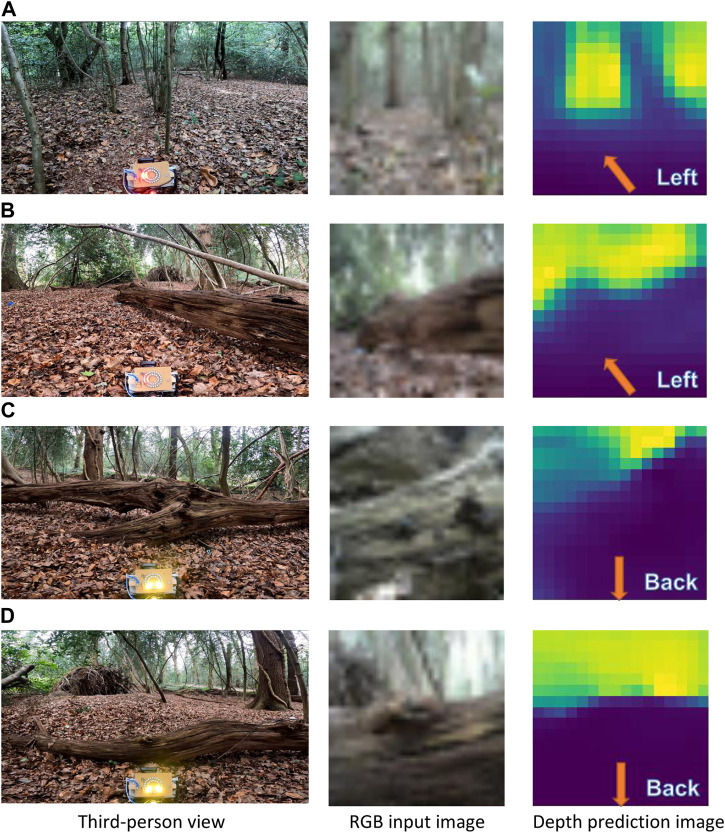
Steering directions navigated by the mobile platform off-trail at the Southampton Common woodland to avoid a number of slender trees **(A)**, and fallen tree trunks and branches **(B–D)**. The steering directions are annotated on the predicted depth image, upsampled here by a factor of two for visual clarity.

Experiments were performed ten times in the forenoon, midday, afternoon, and near sunset, under weather conditions of cloudy, scattered clouds, mostly clear and sunny (see details in Supplementary Table S3 of the Supplementary Material). Across all experiments, the mobile platform was able to reach the goal-way point without sustaining any collisions and incurring any false-negative incidents, irrespective of the time of day and weather conditions. The platform traversed an average distance of 14 ± 6 m from start-to-goal, with a turning rate of 0.72 ± 0.08, in 5.0 ± 2.4 min (see Table 4); a higher turning rate, compared to the forest trail environment, may be due to their being more obstacles off-trail. Despite the higher density of obstacles, the mobile platform was able to avoid them with a sequence of turning actions (see examples in Figures 12A,B of the platform avoiding a slender tree and a fallen tree trunk). Moreover, as with the forest off-trail experiments, the “Go-back” action was accurately triggered to avoid potential collisions (see Figures 12C,D of a fallen tree trunk and a large fallen branch). The “Go-back” action was unnecessarily triggered only once—a false-positive incident—when the platform was facing an incline. Finally, in all experiments in Site B (start waypoint at (5056.1814 N, 124.0148 W)), the platform had to be lifted over an obstacle once; dense bushes on either end of a large fallen tree were blocking all traversable paths to reach the goal.

**TABLE 4:** The trajectory length, turning rate, traversal time and the number of true-positive incidents—the “Go-back” action is accurately triggered on encountering an obstacle—when navigating from start-to-goal off-trail in the Southampton Common woodland. Experiments in site A and site B had start waypoints at (5056.1906 N, 124.0071 W) and (5056.1814 N, 124.0148 W), respectively, and shared a common goal waypoint at (5056.1853 N, 124.0143 W). The experiment weather conditions and times of day are detailed in Supplementary Table S3 of the Supplementary Material.

	Trajectory length (m)	Turning rate	Traversal time (mins)	True-positive “Go-back” actions
Site A				
Run 1	8	0.76	2.6	0
Run 2	10	0.80	3.7	0
Run 3	13	0.62	2.5	0
Run 4	9	0.74	2.7	0
Run 5	10	0.75	3.9	2
Site B				
Run 6	24	0.73	9.3	7
Run 7	17	0.74	6.3	6
Run 8	23	0.55	5.8	6
Run 9	11	0.82	5.2	5
Run 10	19	0.72	8.2	7

## 4 Discussion

In this study, we have implemented a low-viewpoint navigation algorithm for inexpensive small-sized mobile platforms navigating forest environments. For navigation, a depth prediction model was trained to predict depth images from RGB images of a monocular camera mounted on the mobile platform. Subsequently, a simple steering algorithm used predicted depth values to direct the platform towards open traversable areas of the forest, while avoiding obstacles. Our algorithm was extensively tested both in high-fidelity simulated forests, and real-world forests under several different weather conditions and times of day. In field experiments, using no more than a 16 × 16 depth image predicted from a 32 × 32 monocular RGB image, our mobile platform was able to successfully traverse a total of over 750 m of forest terrain comprising small shrubs, dense bushes, tall grass, fallen branches, fallen tree trunks, ditches, mounds and standing trees.

A computational bottleneck of our navigation algorithms is depth prediction, requiring around 0.8 s per RGB image with a DenseNet-121 network on a Raspberry Pi 4. The runtime for depth prediction may be improved with alternative state-of-the-art light-weight convolutional neural network architectures. For instance, the MobileNet (Howard et al., 2017), MobileNet-v2 (Sandler et al., 2018), NASNetMobile (Zoph et al., 2018), ShuffleNet (Zhang et al., 2018), ShuffleNet-v2 (Ma et al., 2018) and the Pyramidal-Depth networks (Poggi et al., 2018) may potentially improve runtime performance, consequent to their small size. However, the performance of these encoders for depth prediction at low viewpoints in forest environments remains to be investigated.

The runtime performance of our algorithms could also be enhanced with marginally more expensive embedded platforms such as the Jetson Nano, Jetson TX1 and Jetson TX2 computers, instead of the Raspberry Pi 4 (Mancini et al., 2018; An et al., 2021; Yang et al., 2021). Our results from a preliminary investigation suggests a three-time increase in average runtime performance for depth prediction of forest scenes at low viewpoints with the Jetson Nano. Finally, budget permitting, the depth prediction model may bes altogether replaced with low-end RGB-D cameras, such as Microsoft Kinect, Intel Realsense, and Orbbec Astra series, thus providing high-resolution depth images, if needed, to our computationally inexpensive steering algorithm.

In challenging forest terrain, the rover may be obstructed by untraversable obstacles, such as dense vegetation, with no accessible paths to reach the destination waypoint. It may therefore be crucial to use strategies to navigate out of the local area. Integrating a robotic arm (Haddeler et al., 2022) on the rover enhances its capabilities for actively exploring such terrain. Equipped with force sensors, the arm may probe its surroundings to identify openings or traversable gaps in the terrain. The additional information may consequently inform the rover’s navigation decisions, improving its ability to maneuver and find suitable paths through the obstacles.

In a few studies, aerial drones are being explored for the monitoring of forest environments (Giusti et al., 2015; Dionisio-Ortega et al., 2018; Iuzzolino et al., 2018; Sudhakar et al., 2020; Zhou et al., 2022). Arguably, rovers capable of navigating forest terrain could complement these technologies. A heterogeneous UGV-UAV team of robots could leverage the strengths of both platforms; the high vantage viewpoint afforded by the aerial drones and the close access to the ground of the rovers. Forest rovers may be adapted to provide a range of ground-based measurements such as physical samples of the soil (Zaman et al., 2022). They could pause to take reliable pollen or greenhouse gas samples close to the ground (Grau Ruiz and O’Brolchain, 2022; Gupta et al., 2022). Moreover, small-sized rovers could be operated quietly, being less intrusive to wildlife than drones (Mulero-Pázmány et al., 2017). Finally, in using drones, the end-user may face a number of hurdles in licensing the vehicles, and be limited to line-of-sight operations (Hodgkinson and Johnston, 2018).

## 5 Concluding remarks

In our study, a mobile platform was pushed by a human operator, following directions provided by the onboard navigation algorithm. Such a platform enabled us to focus solely on the problem of navigation in forest environments, allowing relatively rapid algorithmic prototyping. However, our navigation algorithm has the potential to successfully steer real small-sized rovers. Firstly, the monocular camera mounted inside our mobile platform enclosure is 20 cm off the ground, consistent with the low viewpoint of off-road small-sized rovers (e.g., see rovers deployed in Sebastian et al. (2019); Tang et al. (2019); Murphy et al. (2012), but with expensive sensor hardware and navigating in urban and structured off-road environments). Secondly, our navigation algorithm is robust to motion blur in the RGB images from the movement of our mobile platform, as well as high-contrast lighting, and low-lighting conditions. Finally, our navigation algorithm is tolerant to naturally occurring variations in steering angles and forward displacement step-sizes; these variations are inadvertently caused by the human operator pushing the platform, and from the platform-terrain interactions. Here we report the performance values from the field to give an orientation of how they compare to the simulations. It is important to note that such an approach can only be used to compare the performance of different algorithms if the human operator is neutral or blind to which one is running during the field experiment. The unavoidable bias then applies equally to the techniques under study. In conclusion, the challenges in designing high-endurance low-cost rover hardware capable of self-moving on challenging forest terrain are immense, and are being tackled by us in a separate study (Tarapore et al., 2020); in our future work, we will investigate the performance of our navigation algorithm on real rovers.

## Data Availability

The original contributions presented in the study are included in the article/Supplementary Material, further inquiries can be directed to the corresponding author.
