# Supplementary material for: An embarrassingly simple approach for visual navigation of forest environments
Source: Front Robot AI. 2023 Jun 28;10:1086798. doi: 10.3389/frobt.2023.1086798 (PMC10338120; doi:10.3389/frobt.2023.1086798)
Supplement: Supplementary file 1 [file DataSheet1.pdf]

Supplementary Material - An embarrassingly simple approach for visual navigation of forest environments

1 SIMULATION EXPERIMENTS

Experiments were performed in a 50 m $\times$ 50 m simulated forest with start and goal waypoints at (5,5) and (45,45), respectively.

Table S1. The number of collisions sustained with standing trees when navigating a 50 m $\times$ 50 m simulated forest using ground truth (GT) and predicted depth images (DP) at different resolutions for the steering algorithm.

	16x16		64x48		64x64		128x96		128x128		320x240		Number of obstacles
Trial	GT	DP	GT	DP	GT	DP	GT	DP	GT	DP	GT	DP	
1								1	1		1	1	150
2								1		1		2	156
3	1		1				1						116
4	1							1					154
5													137
6							1					1	166
7			1				1		1		1		148
8		1			1	1	2	1			1		138
9				1							1		140
10					1								116
11											2		179
12													145
13									1		1		94
14							1		1				116
15												1	139
16			2					1		1			226
17		1									1		142
18							1	1					157
19		1											161
20							1					1	161

2 FIELD EXPERIMENTS

A demonstration video of our field experiments is available at `https://www.youtube.com/watch?v=6oZoKS_HeKE`. The forest trail and off-trail experiments in the video were performed on a cloudy day in the afternoon.

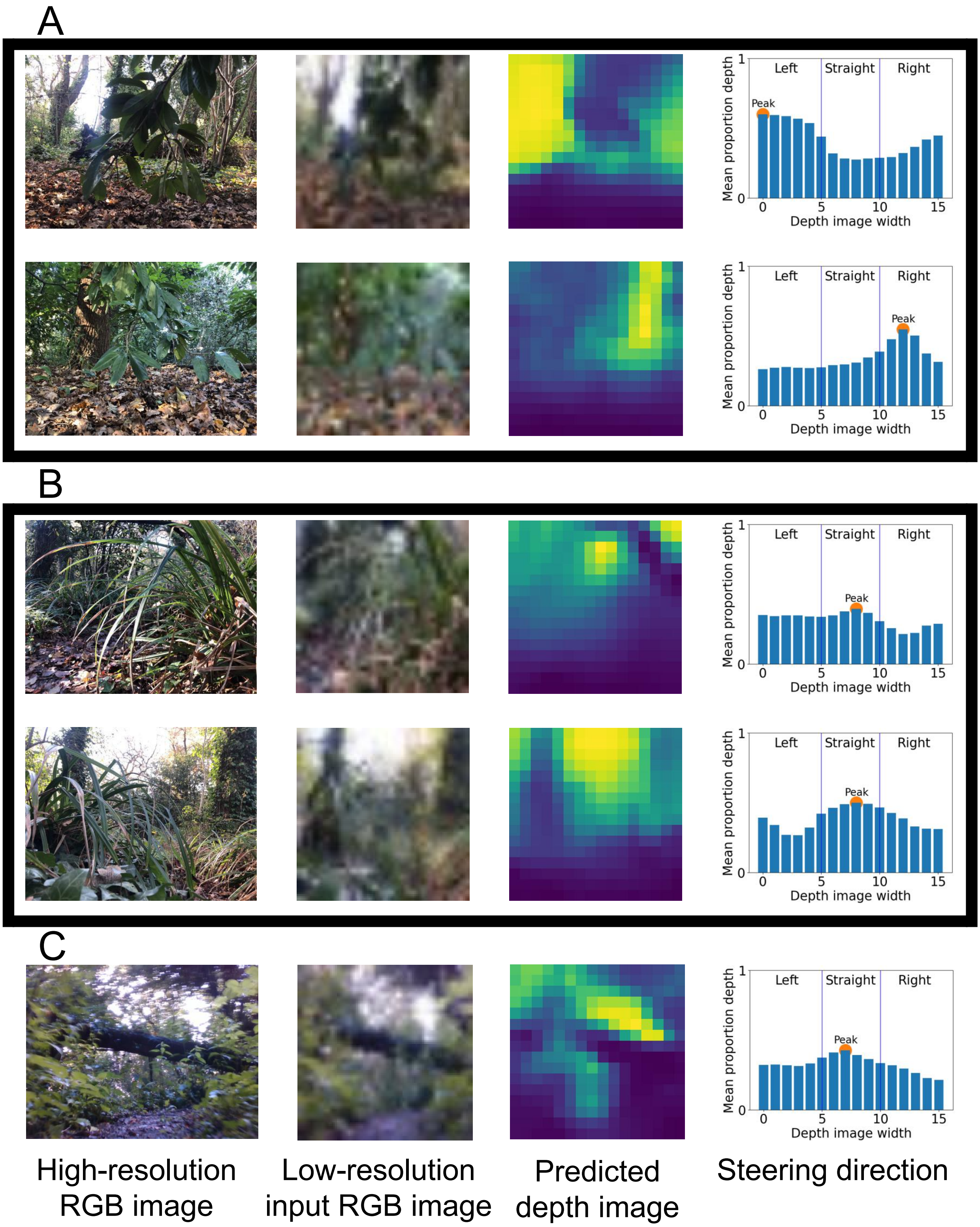

Figure S1. Sample low-resolution 16 × 16 depth images predicted from 32 × 32 input RGB images and corresponding steering directions. The corresponding high-resolution (640 × 480) RGB images are shown for context. The depth prediction model was assessed in a low-hanging branch that yet was not touching the ground (A), traversable tall grass (B) and a high-hanging fallen tree trunk that is not touching the ground (platform can go through the space under the trunk) (C). The displayed predicted depth image has been upsampled by a factor of two for visual clarity.

Table S2. Weather conditions and times of day in following a forest trail from start-to-goal in the Southampton Common woodland.

	Times of day	Weather conditions
Run 1	Forenoon	Sunny, part cloudy
Run 2	Near sunset	Sunny
Run 3	Noon	Clear, sunny
Run 4	Afternoon	Bright, sunny
Run 5	Afternoon	Clear, cloudy

Table S3. Weather conditions and times of day when navigating from start-to-goal off-trail in the Southampton Common woodland. Experiments in site A and site B had start waypoints at (5056.1906 N, 124.0071 W) and (5056.1814 N, 124.0148 W), respectively, and shared a common goal waypoint at (5056.1853 N, 124.0143 W).

	Times of day	Weather conditions
Site A		
Run 1	Forenoon	Clear
Run 2	Noon	Partly sunny
Run 3	Afternoon	Cloudy
Run 4	Afternoon	Mostly clear
Run 5	Near sunset	Scattered clouds
Site B		
Run 6	Midday	Mostly clear
Run 7	Noon	Scattered clouds
Run 8	Forenoon	Partly sunny
Run 9	Near sunset	Clear
Run 10	Afternoon	Cloudy
